# Is It Possible to Detect Return of Spontaneous Circulation during Chest Compression? Evaluation of a Novel Method: Carotid Artery Compression Ultrasound

**DOI:** 10.3390/diagnostics14192213

**Published:** 2024-10-04

**Authors:** Efe Kanter, Ahmet Kayalı, Osman Sezer Çınaroğlu, Adnan Yamanoğlu, Ejder Saylav Bora, Mustafa Agah Tekindal, Mehmet Göktuğ Efgan, Zeynep Karakaya, Fatih Esad Topal

**Affiliations:** 1 Department of Emergency Medicine, Faculty of Medicine, Izmir Katip Çelebi University, 35620 Izmir, Turkey; efekanter@hotmail.com (E.K.); ahmet.kayali083@gmail.com (A.K.); drsezer@hotmail.com (O.S.Ç.); adnanyaman29@hotmail.com (A.Y.); goktugefgan@gmail.com (M.G.E.); zeynep.karakaya@ikc.edu.tr (Z.K.); fatihetopal_18@hotmail.com (F.E.T.); 2 Department of Basic Medical Sciences Biostatistics, Faculty of Medicine, İzmir Katip Çelebi University, 35620 Izmir, Türkiye; mustafaagah.tekindal@ikc.edu.tr

**Keywords:** CPR, ROSC, POCUS-CAC, carotid artery, compression ultrasound

## Abstract

**Objectives:** To evaluate the diagnostic accuracy of carotid artery compression using a point-of-care ultrasound probe (POCUS-CAC) in reducing pulse check times and facilitating the detection of the return of spontaneous circulation (ROSC) during cardiopulmonary resuscitation (CPR) compared to manual palpation (MP). The secondary aim of the study is to assess the ability of POCUS-CAC to detect ROSC during ongoing chest compressions. **Methods:** This prospective study was conducted in a tertiary emergency department between January and June 2023. During CPR, POCUS-CAC was performed by placing a linear ultrasound probe transversely on the lateral neck to assess the compressibility of the carotid artery. Complete compression of the artery without any visible pulsation indicated no ROSC, while resistance to compression or partial compression suggested the presence of ROSC. Simultaneously, another clinician performed manual palpation of the femoral artery. The primary outcome assessed in this study was comparing ROSC detection between POCUS-CAC and traditional methods, and the secondary outcome was comparing the time taken to detect ROSC with each method, and the ability to detect ROSC during ongoing chest compressions. **Results:** The study included 41 cardiac arrest patients and analyzed 496 MP pulse and 1984 POCUS-CAC checks. The mean time to identify a pulse using POCUS-CAC was significantly shorter, at 2.3 (0.5–7.8, SD ± 1.2, 95% CI [2.25, 2.35]) s, compared to 4.7 (2.0–10.5, SD ± 1.8, 95% CI [4.54, 4.86]) s with MP (*p* = 0.004). Additionally, 52.9% of ROSC cases were detected earlier using POCUS-CAC, even during ongoing chest compressions. The sensitivity of POCUS-CAC was 100% (95% CI [80.5–100%]) and the specificity was 87.5% (95% CI [67.6–97.3%]). The POCUS-CAC method required less than 5 s in 99.996% of cases. **Conclusions:** POCUS-CAC significantly reduces pulse check times and enhances the early detection of ROSC during CPR, offering a reliable and rapid alternative to traditional manual palpation methods in emergency settings.

## 1. Introduction

Approximately 7 million people worldwide present to the emergency department each year because of cardiac arrest, accounting for 0.1–0.5% of emergency department admissions [1]. In the management of patients with cardiopulmonary arrest, on the one hand, the detection of underlying effects and identification of correctable causes play the most crucial role in the survival of these patients, while on the other hand, the quality of CPR in this process is one of the most critical parameters in ensuring favorable outcomes and ROSC of patients [2]. Many methods for assessing the quality of CPR have been developed and are under trial worldwide [3], but a gold standard method has not yet been established, and studies on the subject are ongoing.

In CPR management, ECG, end tidal carbon dioxide (EtCO_2_), mean arterial pressure (MAP) measurement, manual pulse palpation, and a combination of these are commonly used to assess the presence of ROSC in the patient and to evaluate the quality of CPR. Despite all these methods, additional methods are needed to evaluate ROSC and the minimum interruption of CPR. Among these additional methods, ultrasonography has been the most prominent in recent years. While ultrasound (US) is used in the evaluation of cardiac contractility, recent studies have used carotid artery pulse to determine CPR quality and ROSC [3,4,5,6,7]. Among these additional methods, point-of-care ultrasound carotid artery compression (POCUS-CAC) has received the most attention recently. One study was conducted by applying compression at certain intervals during CPR [5]. This study claims that applying compression on the carotid artery with a US probe at 30 s intervals makes it possible to determine ROSC, depending on whether the carotid artery is compressed. When applying probe pressure to the carotid artery, an absent pulse was identified through the full compression and absence of pulsation. In contrast, a present pulse was indicated by any visible pulsation or partial compression. According to the claim in this study, this method, unlike other methods, provides continuous monitoring, thus potentially reducing the chest compression interval and contributing to the early detection of ROSC. However, only two studies [4,5] are available in the literature on this topic, and more studies are needed. Both of these studies had small sample sizes and lacked sufficient external validation, which this study aims to address. Additionally, there is a growing need for reliable, real-time methods to detect ROSC during CPR, as existing approaches can lead to delays that impact patient outcomes. This study not only expands the sample size to improve external validation, but also uniquely examines POCUS-CAC’s ability to detect ROSC during ongoing chest compressions, an underexplored area of previous research. By addressing these gaps, this study aims to reduce delays in ROSC detection, improve CPR outcomes, and offer a more comprehensive analysis of the clinical application of POCUS-CAC.

## 2. Materials and Methods

### 2.1. Study Design and Setting

This single-center prospective observational study was conducted in an emergency department in Izmir, Turkey, where 300,000 patients are treated annually, for six months after approval from the IKÇU Clinical Research Ethics Committee (Decision no. 0612, Date: 22 December 2022). Patients were included in the study during working hours when the study team was available. Written informed consent was obtained in the first six hours from the relatives of all patients included in the study. In the critical care unit of the hospital’s emergency department, there are 12 beds where vital signs can be monitored, six mechanical ventilators, two portable, and one fixed ultrasound device. All critically ill patients are brought to the emergency department by an ambulance, and outpatients and cardiac arrest cases are intervened in this area.

### 2.2. Study Population

Patients of both sexes aged ≥ 18 years who presented with in-hospital and out-of-hospital cardiac arrest between January and June 2023 were included in the study. Carotid artery ultrasound (c-US) could not be performed on patients with neck deformities or who had surgery in the neck area. Patients who came to the emergency room after being on CPR for more than 20 min, pregnant patients, and patients who had been seriously injured were also not included in the study

### 2.3. Study Protocol

Before the study started, a team of at least nine members was formed, comprising three emergency medicine faculty members and six senior emergency medicine residents. This team was tasked with performing POCUS and manual pulse checks. All team members received 30 min of carotid artery US training from a faculty member who had been an ultrasound instructor for approximately 13 years.

According to the daily working order, three faculty members from this team were in the hospital throughout the day. At the same time, at least two senior residents were assigned to the critical care area. They were ready to participate in the study in case of a possible cardiopulmonary arrest. With the announcement of a patient’s arrival due to cardiopulmonary arrest in the emergency department or by ambulance, the study team immediately gathered in the relevant area. During each resuscitation, three team members only participated in the pulse check phase.

**Figure 1:**
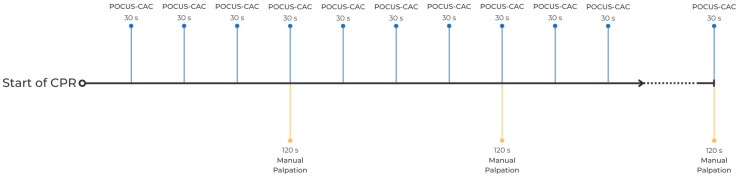
POCUS-CAC protocol.

The patients who experienced cardiopulmonary arrest either before arriving at the emergency department or while in the emergency department were included in the study. A team was established to administer CPR according to the 2023 ESC guidelines, with chest compressions at a rate of 100–120 per minute. This team operated under the supervision of one of three emergency medicine faculty members trained and experienced in CPR. The team members and leader were blinded to each other’s use of equipment and decisions. The first pulse check after the setup of the intubation and capnography equipment was considered the start of the study. All cases were immediately connected to the LIFEPAK 20e defibrillator (Stryker, Kalamazoo, MI, USA), and an endotracheal airway was established for non-intubated patients. An EMMA capnometer (Masimo Corporation, Irvine, CA, USA) was attached to the endotracheal tube used for this airway. During CPR, defibrillation was performed when a shockable rhythm was observed. Cycles with shockable rhythms were recorded, and pulse checks during these cycles were excluded from the calculations. For pulse checks, while one clinician manually checked the pulse over the femoral artery, another simultaneously performed a pulse check over the patient’s carotid artery using a 7–11 MHz linear probe of the Mindray M5 ultrasound system (Medical International Limited, Shenzhen, China) by applying compression. The CPR team leader made the final decision by assessing the rhythm on the monitor, as well as conducting manual femoral pulse checks, ETCO2, and, if necessary, a cardiac ultrasound through the subxiphoid window using a Philips Affinity 70 model (Philips, Andover, MA, USA) with a 2–5 MHz curvilinear probe. Pulse check via manual palpation (MP) of the femoral artery was used as the reference standard, and this was performed every 2 min to check for spontaneous circulation (ROSC) return. Additionally, as soon as resuscitation began, a trained clinician, separate from the team leader, used a carotid ultrasound probe to apply pressure on the carotid artery every 30 s to determine whether the artery could be compressed, which served as the index test (POCUS-CAC) in this study (Figure 1). This frequent monitoring was chosen to capture real-time changes in ROSC status, aiming for greater diagnostic accuracy compared to longer intervals.

In addition to the standard MP method, the team leader used EtCO_2_ values and cardiac ultrasound imaging to determine ROSC. The team leader independently declared the presence or absence of ROSC, separate from the ultrasound operator. Following the POCUS-CAC method [5] in the past, the independent ultrasound operator would check whether there was a pulse when the carotid artery was fully compressed. In cases where the carotid artery was partially compressed or pulsation was observed, ROSC was present. To assist the team leader in making the final decision to terminate CPR, evaluations of EtCO_2_ values and cardiac ultrasound were performed to assess the presence of cardiac contractions. Another senior resident doctor on the study team documented the decision-making process, the EtCO_2_ levels, the rhythm seen on the monitor, the outcomes of the cardiac ultrasound, and the team leader’s final decision in the case report form during each pulse check. CPR was continued for at least 30 min until ROSC was achieved or an exitus decision was made. Upon completion of the CPR process, the patient’s demographic characteristics, chronic diseases, medications, and the causes of death for discharged patients were recorded in the case report form.

### 2.4. Measurement Method: POCUS-CAC

**Figure 2:**
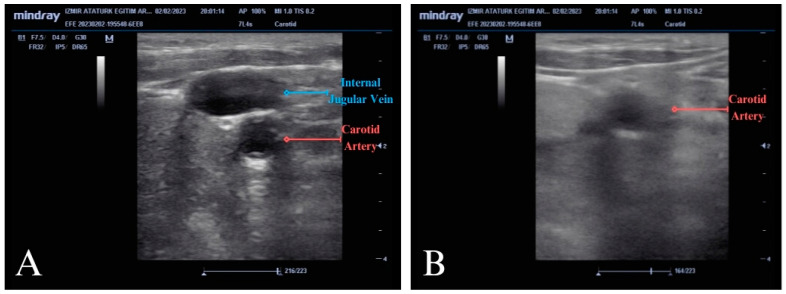
Ultrasound images demonstrating the POCUS-CAC method. (**A**) Internal jugular vein and carotid artery before applying compression. (**B**) The image of the carotid artery that does not collapse under POCUS-CAC during chest compressions (early detection of ROSC).

In this study, the protocol referred to as the POCUS-CAC protocol, previously described by Kang and colleagues, was applied [5]. In this method, during CPR, a linear probe was placed transversely between the two heads of the SCM muscle on the lateral side of the patient’s neck. After visualizing the internal jugular and carotid artery, pressure was applied with the probe until the internal jugular vein was fully compressed. Using this method, the carotid artery behaving like the vein and becoming fully compressed with the absence of pulsation was considered the absence of a pulse. The failure of the carotid artery to compress or the presence of any pulsation after compression was accepted as the presence of a pulse (ROSC) (Figure 2A,B; Video S1).

### 2.5. Data Collection

The data collected for the patients included in the study comprised their age and gender, medical history, probable causes of cardiopulmonary arrest, the location of the arrest (in-hospital/out-of-hospital), the duration of CPR performed in the emergency department, and the outcomes at the end of CPR. Additionally, every 2 min during CPR, it was noted whether ROSC was detected using the MP method, how many seconds it took to make this decision, and whether ROSC was detected using the POCUS-CAC method every 30 s, along with the decision-making time. Furthermore, EtCO_2_ values were recorded every 2 min to assess CPR quality and the presence of ROSC.

The clinicians collecting the data recorded their findings independently and in a blinded manner.

### 2.6. Outcomes

#### 2.6.1. Primary Outcome: Diagnostic Test Accuracy

The primary outcome of this study is comparing the accuracy of diagnostic tests for the detection of the presence or absence of ROSC, checked every two minutes, using the formal method of manual palpation, EtCO_2_, and echocardiography, with the detection using carotid ultrasound.

#### 2.6.2. Secondary Outcome: Time to Detection and Early ROSC Identification

The secondary outcome of the study is the time taken to detect ROSC. The study compares the time taken to detect ROSC using the formal method, measured every two minutes, with the time taken using a compression ultrasound. The secondary outcome of the study is whether ROSC is detected earlier with POCUS-CAC. Additionally, as a method that is rarely reported in the literature, this study aims to determine whether ROSC can be detected during ongoing chest compressions using the POCUS-CAC method.

### 2.7. Sample Size Considerations

A power analysis was conducted for this study, which indicated that 24 patients would be sufficient (with 90.25% sensitivity and 90.01% specificity) to achieve statistical significance. A total sample size of 41 achieves 81.45% power to detect a change in sensitivity from 0.5 to 0.05 using a two-sided binomial test, and 99.8% power to detect a change in specificity from 0.5 to 0.05 using a two-sided binomial test.

### 2.8. Statistical Analysis

All analyses were performed using SPSS (version 26.0; IBM Corp., Armonk, NY, USA). Descriptive statistics were used to summarize patient characteristics. Continuous variables were presented as means ± standard deviations or medians with interquartile ranges, depending on distribution. Categorical variables were presented as frequencies and percentages.

To compare the time to detect ROSC between POCUS-CAC and manual palpation (MP), paired *t*-tests or Wilcoxon signed-rank tests were used, based on data distribution. Categorical variables were compared using the chi-square test (χ^2^).

Diagnostic test accuracy was evaluated by calculating sensitivity, specificity, positive predictive value (PPV), and negative predictive value (NPV) for POCUS-CAC, with 95% confidence intervals (CIs) for each.

A random-effects model was considered due to the multiple pulse checks carried out per patient, but initial analyses showed no significant within-patient variation. A *p*-value of less than 0.05 was considered statistically significant.

## 3. Results

### 3.1. General Characteristics of the Study Population

**Figure 3:**
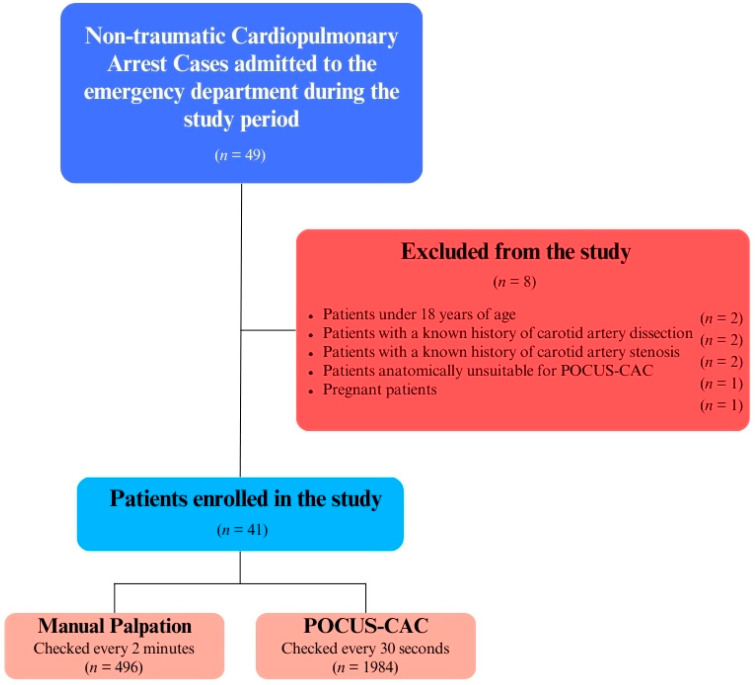
Study population.

A total of 49 non-traumatic cardiac arrest cases, both in-hospital and out-of-hospital, who presented to the emergency department during working hours between January 2023 and June 2023, were included in the study. Of these, patients under 18 years of age (*n* = 2), those with a known history of carotid artery dissection or carotid artery stenosis (*n* = 4), patients anatomically unsuitable for POCUS-CAC application (*n* = 1), and pregnant patients (*n* = 1) were excluded, leaving a total of 41 patients included in the study (Figure 3).

Of the 41 cardiac arrest cases, 19 (46.3%) occurred in-hospital, and 22 (53.7%) were out-of-hospital cardiac arrest cases. The average age of the patients was 71 years, with 24 (58.5%) being females. The most common cause of arrest was cardiogenic, accounting for 36.6% (*n* = 15) of the cases. Following the intervention, 58.5% (*n* = 24) of the patients were pronounced deceased (Ex), while 41.5% (*n* = 17) achieved ROSC. The average duration of CPR was 24.24 min, with intervention times ranging from a minimum of 4 min to a maximum of 44 min. The general characteristics of the patients are shown in Table 1.

### 3.2. Diagnostic Accuracy of POCUS-CAC for ROSC Detection

As shown in Table 2, in the overall measurements, based on the clinical return of circulation decision, 17 of the 41 CPR cases were classified as ROSC, and 24 were classified as exitus. However, according to the POCUS-CAC method, 20 patients were identified as ROSC and 21 as exitus. In three cases, although the clinical return decision was exitus, the inability to compress the carotid artery led to the consideration of ROSC (false positives).

As stated in Table 3, the data show that in the general measurements, the sensitivity of the POCUS-CAC method is 100%, its specificity is 87.5%, the positive predictive value is 85%, and the negative predictive value is 100%.

### 3.3. Analysis of Pulse Check Durations: POCUS-CAC vs. Manual Palpation

In 41 cardiac arrest cases, 496 pulse checks were analyzed using manual palpation (MP) every two minutes, and 1984 pulse checks were analyzed using POCUS-CAC every 30 s.

The mean time to complete a pulse check with manual palpation was 4.7 (2.0–10.5, SD ± 1.8, 95% CI [4.54, 4.86]) s. At the end of CPR, the decision for ROSC or final termination was made in 4.9 (3.0–9.5) s using manual palpation. With POCUS-CAC, the mean time to complete a pulse check was 2.3 (0.5–7.8, SD ± 1.2, 95% CI [2.25, 2.35]) s, which was, on average, 0.49 times earlier than MP (*p* = 0.004). At the end of CPR, the decision for ROSC or final termination was made in 2.1 (0.6–5.2) s using POCUS-CAC, which was, on average, 0.43 times earlier than MP (*p* = 0.009) (Table 4).

In the POCUS-CAC method, only 8 out of 1984 pulse checks (0.004%) took longer than 5 s, whereas in the MP method, 208 out of 496 pulse checks (41.9%) exceeded 5 s *(p* = 0.001).

In 9 out of 17 patients with ROSC (52.9%), ROSC was detected earlier using the POCUS-CAC method while chest compressions were still ongoing during CPR. On average, ROSC was identified 93.33 s earlier in these nine patients with the POCUS-CAC method (Table 4).

**Figure 4:**
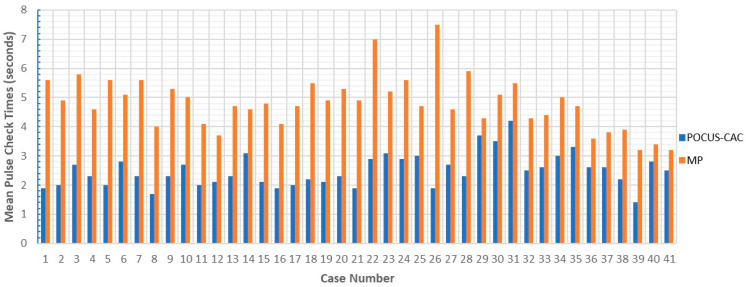
Case—mean pulse check times.

The mean pulse check times for each patient are presented in Figure 4.

### 3.4. Comparison of EtCO_2_ Levels between Exitus and ROSC

There is a statistically significant difference between the EtCO_2_ levels in the exitus and ROSC categories (*p* = 0.001). The average EtCO_2_ level in individuals with ROSC (31.52) is higher compared to the average EtCO_2_ level in individuals classified as exitus (7.45) (Table 5).

## 4. Discussion

Deciding whether a patient achieved a return of spontaneous circulation during cardiopulmonary resuscitation is still time-consuming and can temporarily disrupt the patient’s perfusion [8]. In situations where the full return of circulation has not been achieved, interrupting CPR to check for a pulse is undesirable. However, no standard objective assessment tool has been available to assist with manual palpation or the clinical decision of the team leader. This study demonstrates that POCUS-CAC has high diagnostic accuracy in detecting the presence or absence of ROSC, with a sensitivity of 90.25% and a specificity of 90.01%, providing a reliable and rapid method that can be used without interrupting compressions compared to manual palpation.

A study by Adedipe shows that the ultrasound assessment of common carotid artery blood flow during CPR is possible, but further research is needed to link it to patient outcomes [9]. Moreover, Koch et al. found that carotid ultrasounds performed during CPR can detect irregularities in blood circulation, which could affect the results of CPR [10]. These studies show that the carotid artery US, but not the compression technique, was studied previously.

POCUS utilization during cardiac arrest resuscitation was linked to a notable extension in the duration of pulse checks, approximately doubling the maximum permitted time of 10 s as stated in current guidelines. Acute care providers must carefully monitor the length of breaks in chest compressions while utilizing POCUS during cardiac arrest resuscitation [11]. In this study, out of a total of 1984 ROSC decisions assessed every 30 s using POCUS-CAC, only 8 (0.004%) took 5 s or longer, whereas 208 out of 496 ROSC decisions (41.9%) assessed every two minutes using manual palpation took 5 s or longer. This highlights the necessity of the POCUS-CAC method in CPR management, where time is critical, and every second lost increases the risk of mortality.

Only two studies in the literature examine the relationship between carotid artery compression and ROSC [4,5]. Unlike the other two studies, this study has a larger patient population and systematically focuses on the detectability of ROSC while chest compressions are ongoing. In this study, ROSC was assessed every 30 s using the POCUS-CAC method during ongoing chest compressions, and ROSC was detected in some patients during compressions. This finding suggests that with more studies like this in the future, the ability to detect ROSC without interrupting chest compressions could lead to more effective CPR management and lower mortality rates.

In our study, ROSC was anticipated in nine out of the seventeen ROSC cases using the POCUS-CAC method while chest compressions were still ongoing. The decision for ROSC was made when the carotid artery did not collapse despite the pressure applied through the probe, indicating ROSC without the need to pause compressions. This was later confirmed during the standard CPR protocol when a manual pulse check was performed. This innovation could change CPR management in the future by allowing for ROSC detection without interrupting chest compressions. However, more studies using this method must be widely recognized.

Additionally, in three patients, although ROSC was initially suspected using POCUS-CAC during compressions, the final determination was that there was no pulse. This may have been due to unknown carotid artery calcification in the patient or challenges in applying ultrasound during compression. Therefore, further validation studies and investigations in different settings are needed to understand better the outcomes associated with POCUS-CAC.

Despite the importance of early pulse detection, various studies have shown that healthcare workers need better accuracy rates and take longer to make pulse decisions when detecting central pulses through palpation [12,13]. This poor accuracy highlights the need for improvements in this critical aspect of CPR management. Our study represents a positive step towards strengthening this weaker area of CPR management.

In different studies investigating ROSC detection using ultrasound, one study comparing manual palpation with carotid ultrasound (c-US) found that the average time to make an ROSC decision using manual palpation (MP) was 10.76 ± 1.03 s. In contrast, the c-US method allowed for a decision to be reached in an average of 4.76 ± 2.19 s [14]. Similarly, in another study where the average time to make an ROSC decision using MP was as short as 3 ± 0.17 s, the decision time was reduced to 2.8 ± 0.15 s when using carotid ultrasound (c-US) [7]. Another study showed that only 34 out of 206 pulse decisions made using manual palpation (MP) were completed in under ten seconds (16.5%) [6]. Additionally, studies report that the average time for making an ROSC decision using MP was 6 s, with 76% of the decisions made in under 10 s [15].

In a case series, the POCUS pulse check method was applied to four cases, demonstrating that pulse checks could be completed in under 5 s, suggesting that it could be an alternative to MP3. As far as we know, in the first study where the POCUS-CAC method was named, the average time for pulse checks was 3.50 (2.99–4.99) s using MP, while it was 1.62 (1.14–2.14) s with the POCUS-CAC method. Similarly, in this study, the average pulse check time was 4.7 (2.0–10.5) s using MP, whereas, with the POCUS-CAC method, it was completed in 2.3 (0.5–7.8) s on average (2.4 s and 0.49 times faster than MP (*p* = 0.004)).

In the literature, other studies have also examined EtCO_2_ values as a parameter to support the ROSC decision. According to one study, EtCO_2_ values measured during CPR below 10 mm Hg were generally associated with poor outcomes and low ROSC rates [16]. Crickmer et al. found that an EtCO_2_ value exceeding 20 mm Hg measured 20 min after intubation was a good indicator of ROSC [17]. In another study comparing the effectiveness of capnography during CPR, it was found that the average EtCO_2_ value in the group of patients who expired (19.1 ± 7.8 mm Hg) was significantly lower than in the ROSC group (26.3 ± 6.5 mm Hg). On the other hand, they also claimed that carotid blood flow monitoring during CPR is less helpful than EtCO_2_ in proving the effectiveness of CPR [18]. Similar results were observed in our study, where EtCO_2_ values were measured at each pulse check and at the end of CPR (*n* = 496). The average EtCO_2_ value in patients where ROSC was not achieved was 7.45 ± 2.84 mm Hg, while in patients who achieved ROSC, the average EtCO_2_ value was measured at 31.52 ± 7.18 mm Hg (*p* = 0.001).

## 5. Limitations

A significant portion of the cardiac arrest cases in this study (53.7%) consisted of out-of-hospital cardiac arrest (OHCA) cases. The duration of the CPR administered before the patients were brought to the emergency department could have affected the study results.

The use of ultrasound during CPR requires prior preparation, such as keeping the device continuously on and ready in the field. Although our team was prepared for every cardiac arrest case, in the busy environment of an emergency department, accessing, transporting, turning on, and preparing the US device could take additional time, which may not align with practical scenarios.

In cases where multiple arrests co-occurred in the emergency department, the second team could not access the US device due to its singularity and difficulty transporting it. As a result, these cases were not included in the study.

In our analysis, we categorized the cases based on CPR duration. Since the expectation of ROSC is lower after 30 min compared to the initial minutes, only 1 out of the 11 cases in this timeframe was identified as ROSC based on clinical decision. However, using POCUS-CAC, two cases were identified as ROSC, and due to there being only one false positive, the positive predictive value was calculated at 50%. Similar studies could be conducted in the future with larger patient groups to prevent such misleading results.

## 6. Conclusions

This study demonstrates the diagnostic accuracy of POCUS-CAC in detecting both the presence and absence of ROSC in patients undergoing CPR and compares the methods used to achieve this. It has been clinically and statistically demonstrated that using POCUS-CAC for pulse checks instead of the traditional manual palpation method allows for faster decision-making and provides a reliable alternative in terms of accuracy. By detecting the absence of ROSC early on, this method minimizes interruptions in CPR, thereby better preserving neurological survival.

Moreover, this study has shown that ROSC can be detected in patients even while chest compressions are ongoing using the POCUS-CAC method, and the feasibility of this approach has been confirmed. If this method becomes more widely adopted and developed, it could enable continuous chest compressions until ROSC is detected, improving the resuscitation process. This finding supports the potential for future CPR management without interruptions in compressions, which may lead to higher diagnostic accuracy and improved resuscitation quality. Further studies are necessary to validate these results, which could alter the resuscitation algorithm.

## Figures and Tables

**Table 1:** Study population general characteristics.

		*n*	%
Cause of Arrest	Cardiogenic	15	36.6
	Metabolic	5	12.2
	Pulmonary Embolism	4	9.8
	Septic Shock	5	12.2
	Respiratory Failure	12	29.3
IHCA/OHCA	IHCA	19	46.3
	OHCA	22	53.7
Gender	Female	24	58.5
	Male	17	41.5
Diabetes Mellitus	No	27	65.9
	Yes	14	34.1
Hypertension	No	18	43.9
	Yes	23	56.1
Coronary Artery Disease	No	28	68.3
	Yes	13	31.7
Congestive Heart Failure	No	36	87.8
	Yes	5	12.2
Chronic Kidney Disease	No	32	78.0
	Yes	9	22.0
Malignancy	No	26	63.4
	Yes	15	36.6
Outcome	Exitus	24	58.5
	ROSC	17	41.5
	Total	41	100.0

**Table 2:** Clinical return of circulation decision and POCUS-CAC.

		Clinical Decision	
		Exitus	ROSC
**POCUS-CAC**	Exitus	21	0
	ROSC	3	17

**Table 3:** POCUS-CAC criterion validity.

Method	Sensitivity (%95 CI)	Specificity (%95 CI)	Accuracy	Prevalence	Positive Predictive Value	Negative Predictive Value
**POCUS-CAC**	100% (80.5–100%)	87.5% (67.6–97.3%)	92.7%	41.5%	85.0%	100%

**Table 4:** Analysis of pulse check data.

	MP	POCUS-CAC	Time Difference	*p* Value
Total pulse checks analyzed	496	1984	-	-
Mean time for a pulse check	4.7 s	2.3 s	2.4 s	0.004
	(2.0–10.5)	(0.5–7.8)	(0.49 times earlier)	
Mean time to decide ROSC or termination at the end of CPR	4.9 s	2.1 s	2.8 s	0.009
	(0.5–7.8)	(0.6–5.2)	(0.43 times earlier)	
Number of pulse checks lasting over 5 s (%)	208/496	8/1984	-	0.001
	(41.9%)	(0.004%)		
Number of patients with ROSC detected during chest compressions	-	9/17	93.33 ± 62.65 s	0.001
		(52.9%)	earlier	

**Table 5:** Comparison of exitus/ROSC categories based on EtCO_2_ levels.

	Exitus	ROSC	Test Statistics	*p*
**EtCO_2_**	7.45 ± 2.84	31.52 ± 7.18	−5.301 ^‡^	0.001 ^†^

Numerical variables are presented as mean ± standard deviation. ^‡^: Independent samples *t*-test; ^†^: Mann–Whitney U test.

## Data Availability

All the data for this study are presented in the published article. Upon a reasonable request, any further details are available from the corresponding author (Ejder Saylav Bora ejdersaylav.bora@ikc.edu.tr).

## Supplementary Materials

The following supporting information can be downloaded at: https://www.mdpi.com/article/10.3390/diagnostics14192213/s1, Video S1: Ultrasound Video Demonstrating the POCUS-CAC Method: A Carotid Artery That Does Not Collapse Despite Compression During Ongoing Chest Compressions (Sign of ROSC).
